# Establishment of a brain tumor consortium of Africa: Advancing collaborative research and advocacy for brain tumors in Africa

**DOI:** 10.1093/noajnl/vdae198

**Published:** 2024-11-16

**Authors:** Lateef A Odukoya, Kwadwo Darko, Francis Zerd, Nathalie C Ghomsi, Gloria Kabare, David O Kamson, Jeanette E Eckel-Passow, Robert B Jenkins, Gaspar J Kitange, Andrea O Akinjo, Kabir B Badmos, Olufemi Bankole, Olufemi E Idowu, Claire Karekezi, Elias Edrick, Chukwuyem Ekhator, Victoria M Katasi, Desmond A Brown, Jason Huse, Henry Llewellyn, Margreth Magambo, Michael Magoha, Umaru Barrie, Advera Ngaiza, Arsene D Nyalundja, Minda Okemwa, Lawrence Osei-Tutu, Bernard Petershie, W Elorm Yevudza, Charles C Anunobi, Liadi Tiamiyu, Gbetoho Fortuné Gankpe, Kashaigili Heronima, Dominique Higgins, Kristin Schroeder, Teddy Totimeh, James Balogun, Beverly Cheserem, Arnold B Etame, Ekokobe Fonkem

**Affiliations:** Department of Laboratory Medicine and Pathology, Mayo Clinic, Rochester, New York, USA; Department of Neurosurgery, Korle-Bu Teaching Hospital, Accra, Ghana; Mortuary and Forensic Department, Benjamin Mkapa Hospital, Dodoma, Tanzania; Department of Neurosurgery, Université Félix Houphouet Boigny, Abidjan, Côte d’Ivoire; Neurosurgery Department, Aga Khan University Hospital, Nairobi, Kenya; Department of Oncology and Neurology, Johns Hopkins School of Medicine, Baltimore, Maryland, USA; Department of Quantitative Health Sciences, Mayo Clinic, Rochester, New York, USA; Department of Laboratory Medicine and Pathology, Mayo Clinic, Rochester, New York, USA; Division of Neuro-Oncology Research, The Hormel Institute, University of Minnesota, Austin, Minnesota, USA; Anatomic and Molecular Pathology, Lagos University Teaching Hospital, Lagos, Nigeria; Anatomic and Molecular Pathology, Lagos University Teaching Hospital, Lagos, Nigeria; Neurosurgery Unit, Department of Surgery, College of Medicine, University of Lagos, Lagos, Nigeria; Neurosurgery Unit, Department of Surgery, Lagos State University Teaching Hospital and Lagos State University College of Medicine Ikeja, Lagos, Nigeria; Neurosurgery Unit, Department of Surgery, Rwanda Military Referral and Teaching Hospital, University of Rwanda, Kigali, Rwanda; Department of Pathology, Catholic University of Health and Allied Sciences, Mwanza, Tanzania; Department of Neuro-Oncology, New York Institute of Technology, College of Osteopathic Medicine, New York, New York, USA; Department of Pediatric Oncology and Hematology, Mulago National Referral Hospital, Mulago, Uganda; Department of Neurological Surgery, Mayo Clinic, Rochester, Minnesota, USA; Department of Pathology, University of Texas MD Anderson Cancer Center, Houston, Texas, USA; Department of Global Health & Social Medicine, King’s College London, London, UK; Department of Radiology, Bugando Medical Centre, Mwanza, Tanzania; Department of Surgery, University of Nairobi, Nairobi, Kenya; Department of Neurosurgery, NYU Grossman School of Medicine, New York, New York, USA; Department of Anatomical Pathology, Muhimbili National Hospital, Muhimbili, Tanzania; Center for Tropical Diseases and Global Health, Faculty of Medicine, Université Catholique de Bukavu, Bugabo, Democratic Republic of Congo; Department of Human Pathology, University of Nairobi, Nairobi, Kenya; Paediatric Oncology Unit, Komfo Anokye Teaching Hospital, Kumasi, Ghana; Department of Pathology, Komfo Anokye Teaching Hospital, Kumasi, Ghana; Department of Neurosurgery, Columbia University Vagelos College of Physicians and Surgeons, New York, New York, USA; Anatomic and Molecular Pathology, Lagos University Teaching Hospital, Lagos, Nigeria; Department of Neurological Surgery, Federal Medical Centre, Abeokuta, Nigeria; Department of Neurosurgery, Centre National Hospitalier Universitaire Hubert Koutoukou, Cotonou, Benin; Department of Oncology, Bugando Medical Centre, Mwanza, Tanzania; Department of Neurosurgery, University of North Carolina at Chapel Hill, Chapel Hill, North Carolina, USA; Pediatric Oncology, Bugando Medical Centre, Mwanza, Tanzania; Department of Surgery, Accra Medical Centre, Accra, Ghana; Division of Neurosurgery, Department of Surgery, College of Medicine, University of Ibadan, Ibadan, Nigeria; Neurosurgery Department, Aga Khan University Hospital, Nairobi, Kenya; Department of Neuro-Oncology, Moffitt Cancer Centre, University of South Florida, Tampa, Florida, USA; Department of Neurology, Medical College of Wisconsin, Milwaukee, Wisconson, USA

**Keywords:** brain tumors, CNS tumor registry, global neurosurgery, molecular subtyping, sub-Saharan Africa

## Abstract

**Background:**

Brain tumors represent a significant global health challenge, with rising incidence and mortality impacting individuals worldwide and contributing to cancer-related morbidity and mortality. In Africa, this burden is exacerbated by limited access to advanced diagnostics, treatment options, and multidisciplinary care, compounded by the absence of standardized cancer registration and tumor biobanking. The introduction of molecular diagnostics, as outlined in the 2021 World Health Organization central nervous system (CNS) tumor classification, adds complexity to brain tumor management, particularly in regions with scarce resources.

**Methods:**

To address these issues, the Brain Tumor Consortium for Africa (BTCA) was established in 2023, bringing together experts to improve CNS tumor diagnosis, patient care, and research. The initial project, conducted via an electronic questionnaire, aimed to assess neuro-oncology capacity across Sub-Saharan Africa.

**Results:**

The study revealed significant gaps, with a limited number of institutions incorporating molecular subtyping into their diagnostic algorithms. The consortium’s efforts focus on enhancing local data use, informing public policy, and promoting collaboration to advance neuro-oncology practices in Africa. By fostering a network enlisting the expertise of collaborators in the fields of neurosurgery, neurology, neuropathology, anatomic pathology, and medical and radiation oncology, the BTCA seeks to improve brain tumor management through better diagnostics, infrastructure, and policy advocacy. Future directions include expanding molecular diagnostic capabilities, standardizing brain tumor biobanking, enhancing data collection, and advocating for improved brain tumor care in national health agendas.

**Conclusions:**

The BTCA represents a pioneering model of collaboration and innovation in addressing the unique challenges of brain tumor care in Africa.

**Key Points**

- Addressing Diagnostic and Treatment Gaps: The BTCA seeks to tackle significant gaps in CNS tumor diagnostics and treatment across Africa. Preliminary findings reveal significant challenges with advanced molecular subtyping, highlighting the need for improved infrastructure and resources to integrate these advancements into patient care.
- Collaborative Approach for Enhanced Care: Established in 2023, the BTCA fosters collaboration among a diverse network of experts and institutions from Africa and beyond. This collaborative effort aims to enhance brain tumor diagnosis, patient management, and research through collective expertise, shared resources, and a focus on improving molecular diagnostics and cancer registries.
- Policy and Research Impact: The consortium’s initiatives are designed to inform public policy and raise awareness about brain tumor care in Africa. By providing evidence-based recommendations and mapping out current neuro-oncology capacity, the BTCA aims to guide policy decisions and advocate for better healthcare practices, ultimately striving to improve patient outcomes and advance neuro-oncology in the region.

**Importance of The Study**

Establishing the BTCA is crucial for addressing the significant challenges in brain and spine tumor care and research across Sub-Saharan Africa. By uniting experts from diverse medical specialties and leveraging collective resources, the BTCA aims to facilitate the advancement of diagnostic and treatment practices, which are often hindered by limited infrastructure and expertise in the region. The consortium’s collaborative approach enhances the ability to share knowledge, develop standardized protocols, and advocate for policy changes, ultimately driving improvements in patient outcomes and advancing neuro-oncology practice. This collaborative framework is essential for overcoming regional disparities and fostering progress in brain tumor management and research.

Brain tumors present a substantial global health burden, with rising incidence and mortality rates affecting individuals across the world.^1^ They are a leading cause of cancer-related morbidity and mortality, encompassing both primary and metastatic tumors.^1^ In Africa, the impact is exacerbated by severely limited access to advanced diagnostic technologies, effective treatment options, and comprehensive care.^2^ The absence of standardized cancer registration systems and tumor biobanking further complicates efforts to understand the epidemiology of brain tumors and to improve patient outcomes.^3^

The challenges posed by brain tumors are magnified by their complexity and the urgent need for innovative treatments. This situation is particularly dire in regions with limited resources, where specialized care is often scarce or unavailable.^4^ The 2021 World Health Organization Central nervous system (CNS) tumor classification introduces advanced molecular diagnostic techniques, which add an additional layer of complexity to the already challenging landscape of brain tumor management in low- and middle-income countries (LMICs).^5^ While recent reviews have laid the foundational understanding of the epidemiological, molecular, and genetic landscape of brain tumors in Sub-Saharan Africa (SSA), translating this evidence into clinical practice is challenging.^6–8^ For instance, the integration of molecular diagnostics into patient care in the region is fraught with difficulties due to a lack of expertise in molecular pathology, insufficient infrastructure, and the high costs associated with these advanced diagnostic tools.^9^

To address these pressing challenges, the Brain Tumor Consortium for Africa (BTCA) was established in 2023. The consortium aims to tackle these issues through a collaborative approach, providing a platform for experts from various medical specialties to come together. By fostering collaboration and leveraging collective expertise, the BTCA seeks to accelerate efforts in improving brain and spine tumor diagnosis, enhancing patient care, and advancing research. The goal is to significantly enhance patient management outcomes in SSA, paving the way for more effective and equitable tumor care in the region.

## Inception of the BTCA

The consortium was formed in 2022 through the collaborative efforts of LAO, a research fellow at Mayo Clinic Rochester, along with EF, RBJ, DHL, GK, and JEP, who together conceived the idea. LAO, under the mentorship of Drs. RBJ and JEP, were investigating molecular features of gliomas in Lagos, Nigeria, which highlighted the need for a collaborative approach to address challenges in CNS tumor diagnosis, cancer registration, and tumor biobanking in Africa. Initial discussions and virtual meetings in 2022, facilitated by the Society for NeuroOncology SSA (SNOSSA), gathered clinical specialists from SSA and North America to discuss consortium objectives and projects. The first physical meeting occurred in Tampa, Florida, at the Society for Neuro Oncology (SNO) Annual Meeting in November 2022, where the group planned its inaugural project on brain tumors in Africa.

## BTCA Vision and Objectives

### Vision.—

The BTCA envisions to become a leading global entity in brain tumor research and advocacy focused on advancing neuro-oncology practice in Africa. Through collaboration, innovation, and shared resources, the consortium seeks to transform the landscape of brain tumor diagnosis, management, and research with a particular focus on molecular testing, cancer registry, and biorepository. The BTCA seeks to realize these visions by leveraging core strengths in soft diplomacy, advocacy, and bridge-building.

### Aspirations and future objectives.

—The BTCA has yet to be formally incorporated, so its guiding principles currently serve as aspirations rather than the concrete objectives of a fully established organization. In summary, the consortium aspires to create a collaborative network for interdisciplinary research, aiming to gather reliable data and facilitate the exchange of between centers that can be used to:

1. Advance strategies towards incorporating molecular testing into CNS tumor patient work-up.
2. Advance strategies for developing CNS tumor-specific biorepositories while securing and managing funding and resources to support ongoing and future research initiatives.
3. Promote efforts ensuring proper CNS tumor registration.
4. Promote efforts for the development of imaging data repositories.
5. Promote education and awareness about CNS tumors and the consortium’s efforts by disseminating knowledge through workshops, conferences, and publications.

## BTCA Activities to Date

### Establishing a platform.—

Africa faces a critical shortage of specialized medical professionals, intensifying the region’s healthcare workforce crisis. SSA, which bears more than 24% of the global disease burden has only 3% of the world’s health workforce.^10^ This disparity is particularly pronounced in fields like neurosurgery, where the availability of trained professionals is critically low.^11^ Addressing this workforce gap requires innovative solutions that go beyond increasing the number of healthcare providers. Auxiliary measures include redefining and creating new infrastructure, training health professionals and paraprofessionals, and harnessing technology—especially telecommunications—to overcome geographic constraints and further effective collaboration.^12^ The BTCA’s preliminary emphasis on multidisciplinary collaboration aligns with global recommendations for improving cancer care in low-resource settings.^12^ It is through such efforts that Africa can begin to build a resilient healthcare workforce capable of meeting the continent’s growing needs in cancer diagnostics and care.

The BTCA has already made significant strides in uniting a substantial portion of the brain tumor research community in Africa. Through this collaborative effort, the consortium created a platform for researchers and clinicians to strategize collectively and develop innovative approaches to advance both clinical practice and research in the region. This collective initiative has also led to the publication of peer-reviewed articles aligned with the consortium’s mission to improve molecular diagnosis of brain tumors. Notably, “Histopathologic and molecular profile of gliomas diagnosed in Lagos Nigeria” by *Odukoya* et al.,^13^ exemplifies the consortium’s commitment to enhancing the understanding and management of brain tumors in SSA. The consortium continues to be active with posters and oral presentations at both the Annual SNOSSA conference in Africa since 2023 and the upcoming SNO 2024 Annual meeting in Houston, TX.

Collaboration is at the heart of the BTCA’s vision, providing a platform for multidisciplinary engagement and the sharing of expertise among neuro-oncology specialists (**Table 1**). Collaborative efforts in research, training, and resources present opportunities to enhance brain tumor diagnostics and neuro-oncology care.^2^ The consortium’s maiden project, which involved a diverse range of specialties including neurosurgery, neurology, neuropathology, and oncology, exemplifies the power of collective action. The participation of over 50 institutions, many of which lack access to advanced molecular diagnostic tools, underscores the value of pooling resources to overcome common barriers. As the BTCA continues to grow, its collaborative framework will remain a cornerstone of its efforts to enhance neuro-oncology practice, research, and policy in Africa.

**Table 1.** Institution and Departments of BTCA Collaborators

Country	Institution	Department
Abidjan, Côte d’Ivoire	Université Félix Houphouet Boigny	Department of Neurosurgery
Accra, Ghana	Korle-Bu Teaching Hospital	Department of Neurosurgery
Accra, Ghana	Accra Medical Centre	Department of Surgery
Austin, USA	The Hormel Institute, University of Minnesota	Division of Neuro-Oncology Research
Bugabo, Democratic Republic of Congo	Université Catholique de Bukavu	Center for Tropical Diseases and Global Health, Faculty of Medicine
Cotonou, Benin	Centre National Hospitalier Universitaire Hubert Koutoukou	Department of Neurosurgery
Dodoma, Tanzania	Benjamin Mkapa Hospital	Mortuary and Forensic Department
Florida, USA	Moffitt Cancer Centre, University of South Florida	Department of Neuro-Oncology
Ibadan, Nigeria	Department of Surgery, College of Medicine, University of Ibadan	Division of Neurosurgery
Kigali, Rwanda	Rwanda Military Referral and Teaching Hospital, University of Rwanda	Neurosurgery Unit, Department of Surgery
Kumasi, Ghana	Komfo Anokye Teaching Hospital	Pediatric Oncology Unit
Kumasi, Ghana	Komfo Anokye Teaching Hospital	Department of Pathology
Lagos, Nigeria	Lagos University Teaching Hospital	Anatomic and Molecular Pathology
Lagos, Nigeria	Lagos University Teaching Hospital	Neurosurgery Unit, Department of Surgery
Lagos, Nigeria	Lagos State University Teaching Hospital	Neurosurgery Unit, Department of Surgery
London, England	King’s College London	Department of Global Health & Social Medicine
Maryland, USA	Johns Hopkins School of Medicine	Department of Oncology and Neurology
Muhimbili, Tanzania	Muhimbili National Hospital	Department of Anatomical Pathology
Mulago, Uganda	Mulago National Referral Hospital	Department of Pediatric Oncology and Hematology
Mwanza, Tanzania	Catholic University of Health and Allied Sciences	Department of Pathology
Mwanza, Tanzania	Bugando Medical Centre	Department of Radiology
Mwanza, Tanzania	Bugando Medical Centre	Department of Oncology
Mwanza, Tanzania	Bugando Medical Centre	Pediatric Oncology
Nairobi, Kenya	Aga Khan University Hospital	Neurosurgery Department
Nairobi, Kenya	University of Nairobi	Department of Surgery
Nairobi, Kenya	University of Nairobi	Department of Human Pathology
New York, USA	New York Institute of Technology, College of Osteopathic Medicine	Department of Neuro-Oncology
New York, USA	NYU Grossman School of Medicine	Department of Neurosurgery
New York, USA	Columbia University Vagelos College of Physicians and Surgeons	Department of Neurosurgery
North Carolina, USA	University of North Carolina at Chapel Hill	Department of Neurosurgery
Ogun State, Nigeria	Federal Medical Centre, Abeokuta	Department of Neurological Surgery
Rochester, USA	Mayo Clinic	Department of Laboratory Medicine and Pathology
Rochester, USA	Mayo Clinic	Department of Quantitative Health Sciences
Rochester, USA	Mayo Clinic	Department of Neurological Surgery
Texas, USA	University of Texas MD Anderson Cancer Center	Department of Pathology
Wisconsin, USA	Medical College of Wisconsin	Department of Neurology

## Mapping the Terrain of Neuro-oncology in Africa

The maiden study was motivated by the significant lack of detailed knowledge surrounding neuro-oncology capacity across Africa. Previous efforts to assess global molecular diagnostic capabilities, such as the 2016 survey by Andreiuolo et al.,^14^ highlighted the scarcity of data from SSA, with only a single response from SSA (South Africa). This highlights the gap in our understanding of the region’s neuro-oncology resources and infrastructure, which has largely been inferred from anecdotal reports. The BTCA survey represents the first comprehensive assessment of neuro-oncology capacity in Africa. It was made available in English, French, and Swahili, targeting neuro-oncology care practitioners throughout the continent. The primary goals were to map the distribution and capacity of neuro-oncology services and workforce, assess the presence of brain tumor registries and biobanking capabilities, and evaluate the capacity for molecular diagnosis of brain tumors within the region. Interim results from this questionnaire-based survey were presented at the 2024 Annual SNOSSA meeting in Harare Zimbabwe, which brought new visibility to the project and led to additional responses. As of drafting this manuscript, responses are still being accepted, with plans to reach more centers to enhance the comprehensiveness of the results. We are hopeful to share the complete findings with the broader neuro-oncological community in the near future.

This project is especially timely given molecular diagnostics has revolutionized brain tumor treatment by enabling more precise and targeted therapies.^15^ However, implementing these advancements in LMICs remains challenging due to limited resources and infrastructure.^15,16^ The 2021 World Health Organization classification of CNS tumors, which relies heavily on molecular analysis, exemplifies these challenges, as many LMICs experience difficulties in adopting such diagnostic tools.^17^ Additionally, global disparities in precision medicine highlight that the molecular characteristics of tumors can vary by region, yet there is a significant lack of data from LMICs, further widening the gap in effective treatment options.^16^ Although the project focuses primarily on primary brain tumors particularly gliomas, the approach and collaborative network have significant implications for brain metastases as well, particularly those originating from non-small cell lung cancer (NSCLC) (ALK, ROS, EGFR) and breast cancer brain metastases (HER2, ER/PR, TNBC) with actionable mutations where targeted therapies demonstrate strong intracranial response rates.^18,19^ Although exploring the access and capacity for molecular diagnoses of these primary tumors is outside the scope of this study, it is noteworthy that access to targeted therapies for NSCLC and breast cancer can infer access for their corresponding brain metastases and highlights the importance of expanding on the current project in the future.

By mapping out the current neuro-oncology capacity, including workforce distribution, available services, and molecular diagnostic capabilities, the consortium aims to provide evidence-based recommendations to improve neuro-oncology infrastructure. This is particularly important in a region where the burden of brain tumors is compounded by limited resources and expertise.^9^ Our preliminary findings highlight significant diagnostic gaps, with fewer than half of the reported institutions incorporating molecular subtyping into their diagnostic algorithm. While not surprising, these results represent a critical opportunity to enhance brain tumor care in SSA. There is an urgent need to develop infrastructure and resources in SSA to support the adoption of molecular diagnostics in brain tumor treatment. Though challenging, these steps are essential milestones in advancing neuro-oncology in the region.

## Developing Brain Tumor Epidemiology in Africa

Minimal large-scale research has been conducted on the national incidence of brain tumors in Africa.^3^ Although numerous hospital-based studies have attempted to assess the burden of brain tumors, their reliability is limited by their dependence on local hospital registries.^3^ Brain tumor epidemiology in Africa is severely constrained by the challenges associated with maintaining robust cancer registries and the reliance on genetic data predominantly generated from populations outside the continent.^3^ This limited understanding hampers efforts to develop tailored treatment strategies and undermines the ability to address region-specific genetic and environmental factors that may influence tumor biology. The lack of comprehensive local data heightens the stakes for improving neuro-oncology research and care in Africa, where the burden of brain tumors remains inadequately understood.

The implications of diagnostic accuracy in neuro-oncology cannot be overemphasized. One of the central aspirations of the consortium is to establish collaborative biorepositories that span the continent, providing a platform for standardized tumor sample collection, storage, and genomic analysis. This initiative seeks to address current gaps in molecular diagnostics by fostering partnerships that enable the sharing of resources, expertise, and data. By developing such infrastructure, the BTCA aims to enhance the capacity for genomics research, facilitating more precise molecular characterization of brain tumors and improving diagnostic accuracy and therapeutic strategies on the continent. The survey reflects these goals, highlighting the importance of biobanking in building a robust neuro-oncology network for Africa.

The role of research in policymaking has become increasingly vital as the focus shifts to maximizing the value of healthcare expenditures. The primary goal of health-related research is to generate new knowledge that enhances patient and population care.^20^ To fully leverage research evidence, there is a need for better systems to generate, process, and share research findings. Investing in accessible data repositories will make evidence more readily available and useful for policymakers, thereby improving its impact on policy decisions.^20^

## Advancing Local Approaches to Care

Treatment protocols for brain tumors in LMICs are often adapted from those used in high-income countries.^21^ However, implementing these protocols in LMICs presents significant challenges due to differences in resources, healthcare infrastructure, and patient needs. There is an increasing recognition of the need to tailor these protocols to the specific context of LMICs, considering available resources and patient demographics.^21^ To achieve optimal outcomes, this adaptation must be coupled with ongoing local research to advance understanding and maximize the benefits for patients. This highlights the ever-growing need to tailor treatment strategies to local contexts by exploiting local data-sharing capabilities and establishing services, guidance, and policies that are appropriate to existing infrastructures. This approach emphasizes the importance of developing protocols that reflect local realities, ensuring they are feasible and effective in African settings. The BTCA exemplifies the potential of leveraging local data to improve neuro-oncology practices across the continent. A better understanding of regional brain tumor epidemiology, combined with assessing and improving neuro-oncology capacity and establishing a collaborative platform supports the development of evidence-based guidelines that are more appropriate for local conditions to improve patient care.

Nonetheless, engaging local governments is crucial for developing infrastructure and advancing neuro-oncology practices in LMICs. Governments play a key role in addressing healthcare gaps by allocating resources, building capacity, and fostering partnerships. Public policy supports advocacy, raises awareness, and drives investment in essential infrastructure like molecular testing, biobanks, and training programs. We aim to foster collaboration between the BTCA and government entities to bridge research and policy, ensuring that evidence-based solutions are integrated into healthcare agendas. This partnership will be essential for driving sustainable change, enhancing care delivery, and achieving better patient outcomes across the continent.

## Future Directions

Looking ahead, the consortium aims to increase its network within and outside the continent, with hopes of expanding molecular diagnostic capabilities across more institutions, developing and standardizing brain tumor biobanking, and enhancing data collection through integration with national cancer registries. The consortium aims to strengthen collaborative research networks by involving more centers and fostering interdisciplinary initiatives while focusing on capacity-building and training programs for healthcare professionals. Additionally, the BTCA plans to engage in advocacy efforts to influence public policy and drive awareness of brain tumor care, ensuring it is prioritized in national health agendas. Ongoing publication and dissemination of research findings will remain a priority, alongside securing sustainable funding sources to support long-term projects. Albeit huge tasks, these efforts are necessary to improve CNS tumor care.

## Conclusion

The BTCA stands as a pioneering model of collaboration and innovation in the field of CNS tumor research and care across Africa. By uniting a diverse network of experts and leveraging collective expertise, the consortium is strategically positioned to address the unique challenges of CNS tumor diagnosis, treatment, and research in the region. With a focus on advancing molecular diagnostics, enhancing cancer registries, and developing biobanking capabilities, the BTCA is poised to make critical contributions to the understanding and management of brain tumors, ultimately improving the care of brain tumor patients across the continent and beyond. This consensus statement represents the current progress of discussions within the BTCA and serves as an initial step in uniting perspectives across the brain tumor research community in Africa. It reflects the insights gathered so far while acknowledging that more developments and refined analyses are forthcoming. A formal study will follow, building explicitly upon the results of the survey to further explore the consortium’s capacity, challenges, and future directions. This evolving process highlights our commitment to the neuro-oncological field.

## Data Availability

No primary data were generated or analyzed in this study. All data discussed are derived from previously published studies and are publicly available through the sources cited in this manuscript.
